# Organic and psychiatric symptoms of “Long COVID” among Tunisian patients: a cross sectional study

**DOI:** 10.1192/j.eurpsy.2023.504

**Published:** 2023-07-19

**Authors:** F. Zaouali, A. Chaouch, F. Boubaker, S. Bouchareb, H. E. Mrabet, A. Ben Mabouk, I. Touil, L. Boussofara, J. Knani, N. Boudawara, W. Alaya, M. H. Sfar

**Affiliations:** ^1^Endocrinology Depatment; ^2^Pneumology-allergology department, Tahar Sfar University Hospital, Mahdia, Tunisia

## Abstract

**Introduction:**

Long COVID is a condition characterized by long-term health problems persisting or appearing after the typical recovery period of COVID-19. Physical symptoms such as respiratory, neurological and musculoskeletal complaints were initially described in the foreground. A little after, psychological disorders have been widely reported.

**Objectives:**

The aim of this study was to screen for somatic and anxio-depressive disorders of Long COVID.

**Methods:**

A cross sectional descriptive study included the patients consulting within a minimum of 1 month after a COVID-19 infection. It was conducted at Taher Sfar university hospital of Mahdia over a period of one year from March 2020 to March 2021. A questionnaire and physical examination were used to look for physical symptoms and the Hospital Anxiety and Depression scale (HAD) was used to screen for anxio-depressive disorders.

**Results:**

We recruited 137 patients. The median age was of 60 years with a sex ratio M/F at 0.073. Obesity was the most frequent comorbidity (36%) followed by diabetes (35%) and hypertension (32%). More than a quarter of patients was hospitalized (30%) during the acute phase, while the others (70%) were confined at home. The median stay duration at home or hospital was of 10 days with extremes ranging from 0 to 21 days. The most frequent post-COVID symptoms were dyspnea, mood disorders, myalgia, arthralgia, dry cough, sleep disorders and anorexia in 45%, 30%, 30%, 20%, 16%, 15% and 14% of cases, respectively. Pulmonary auscultation was normal in 86% of our patients, for the others we noted crackles, ronchi and wheezing among 9%, 1% and 1% of patients. The median oxygen saturation was 97% with a range from 93 to 99%. The majority of our patients (120) had saturation more than 95% in ambient air. The median HAD score was situated at 19 [8, 33]. Anxio-depressive disorders were present 61% of cases. A severe depression was noted among 24% of patients. and a severe anxiety among 28% of them.

**Conclusions:**

Our study highlighted a high prevalence of anxio-depressive disorders (62%) which exeeds the prevalence described in the literature. The systematic use of the HAD scale among consultants could be the explanation. Thus, psychological screening and support should be considered when managing patients having a history of COVID-19 infection. Citizens should comply with the relevant legal provisions making vaccination compulsory as it was found that COVID-19 vaccination reduced long COVID risk.

**Disclosure of Interest:**

None Declared

